# Study on Interfacial Interlocking Effect of Ultrasonic Vibration-Assisted Adhesive Bonding

**DOI:** 10.3390/polym14132622

**Published:** 2022-06-28

**Authors:** Yunwei Cao, Hui Wang, Qingsong Zhang, Kai Huang, Yizhe Chen, Jinhuo Wang, Fei Yan, Huafeng Liu

**Affiliations:** 1 Hubei Key Laboratory of Advanced Technology for Automotive Components, Wuhan University of Technology, Wuhan 430070, China; caoyw@whut.edu.cn (Y.C.); kaihuang@whut.edu.cn (K.H.); lhf2000@whut.edu.cn (H.L.); 2 Hubei Collaborative Innovation Center for Automotive Components Technology, Wuhan 430070, China; zhangqs@whut.edu.cn; 3 Fujian Key Laboratory of Functional Materials and Applications, Xiamen University of Technology, Xiamen 361024, China; 4 Hubei Research Center for New Energy & Intelligent Connected Vehicle, Wuhan University of Technology, Wuhan 430070, China; fyan001@whut.edu.cn

**Keywords:** ultrasonic vibration, adhesive resin, micromechanical interlocking, molecular dynamics

## Abstract

Carbon fiber reinforced polymer (CFRP) blades are often exposed to wild and even harsh environments. The durability of the blade can be greatly improved by adhesively bonding a Ni erosion shield to the leading edge. In a traditional bonding process, the permeation of adhesive is poor at the interface, which gives an insufficient micromechanical interlocking. In this study, ultrasonic vibration was applied during the bonding process of sandblasted Ni plates and CFRP laminates. The values of shear strength were measured by tensile tests to verify the strengthening effect of applying ultrasonication. The cross-section of the bonded interface was characterized by scanning electron microscopy (SEM) and energy-dispersive spectroscopy (EDS), and the surfaces with different treatments were explored by atomic force microscopy (AFM). The cross-sectional morphology and failure model of the samples were investigated. The strengthening mechanism was then studied by a molecular dynamics method. For the simulation of molecular dynamics, the CFRP/Ni bonding interface model was designed using the Materials Studio software package. The Perl scripts were used to simulate the ultrasonic vibration with different frequencies and amplitudes. The results showed that the ultrasonic process could improve the permeability and uniformity of the adhesive, enhancing the micromechanical interlocking effect.

## 1. Introduction

Carbon fiber reinforced polymer (CFRP) blades are used in wind turbines, aircraft engines and other applications [1,2]. They work at high speed and are often exposed to wild and even severe environments. Despite the excellent characteristics of the material, corrosion and damage are inevitable. In order to protect the blade from lightning strikes, bird strikes or wind erosion [3,4] and reduce maintenance costs, a Ni erosion shield is generally connected to the leading edge in the manufacturing.

Adhesive bonding, as one of the prevailing technologies, is widely used in the connection of the blades and shields. To improve the bonding, surface treatment is required to modify the adherend surface. As a physical surface modification method, sandblasting treatment has been confirmed to adjust the surface roughness via blasting with hard ceramic particles [5]. After the adhesive is applied to the sandblasted surface, micro-level rivets are produced at the bonding interface, providing mechanical interlocking, which can improve the adhesion [6,7]. Nevertheless, in a traditional bonding process like that, the permeation of the adhesive on the pretreated surface is poor and insufficient because of the large viscosity and complex surface status, weakening the interlocking effect, so a number of studies have been focusing on promoting the permeation of adhesive in the bonding process.

In recent years, many studies have shown that the introduction of ultrasonic vibration can strengthen the interfacial interlocking effect. Wen et al. [8] studied the strengthening effect of isotactic polypropylene/aluminum hybrids manufactured by the self-made ultrasonic-assisted hot press molding (UAHPM) technology, and further studied the thermal property and crystal structure. It was found that the formed micromechanical interlocking allowed the hybrids to have stronger bonding strength. Li et al. [9] introduced high-power ultrasonic treatment into a welding process and studied the morphology and mechanical properties of the welded aluminum parts. The results showed that obvious mechanical interlocking was formed at the interface. Wang et al. [10] applied ultrasonic vibration in the bonding process of CFRP laminates pretreated with laser ablation. The results showed that the ultrasonic process promoted the penetration of adhesive and enhanced the anchoring effect. The strengthening effect has been investigated in terms of capillary rise [11], bubble removal [12] and fluidity [13], but there is still a lack of unified and comprehensive explanation of the mechanism. For aerospace [14], thermosonic flip chip (TSFC) [15] and automotive industry [16] applications, the utilization and strengthening mechanism of ultrasonic vibration are essential research areas.

In this study, ultrasonic vibration-assisted adhesive bonding technology was utilized to promote the bonding performance of CFRP/Ni joints. The sandblasting treatment (80# SiC) was used to pretreat the Ni plates. Then, an ultrasonic workbench was used to apply vibration to CFRP laminates during the bonding process and the vibration was transmitted to the adhesive layer through CFRP laminates. The tensile test was chosen to measure the shear strength to verify the strengthening effect. Scanning electron microscopy (SEM) and energy-dispersive spectroscopy (EDS) were used to characterize the cross-section of the joints. Atomic force microscopy (AFM) was used to explore the surface morphology of Ni plates treated by different methods. The cross-sectional morphology and failure mode of the samples were also investigated. Finally, Materials Studio 2019 was used to simulate the ultrasonic process at a molecular level. The strengthening mechanism of the ultrasonic vibration-assisted adhesive bonding was analyzed.

## 2. Materials and Methods

### 2.1. Materials and Surface Treatment

A two-component epoxy adhesive (Loctite 9309, supplied by Loctite Corporation, Rocky Hill, CT, USA) was used in the bonding. When using the adhesive, component A was first added into a glass beaker, and then component B was added in according to a mass ratio of 100:22. To homogeneously mix the adhesive, a glass rod was used to stir the two components for at least one minute at room temperature. Finally, the mixed adhesive was placed in a vacuum oven to remove bubbles inside.

CFRP laminates were manufactured by compression molding using nine layers of prepregs (T300-3K, twill woven carbon fiber cloth, matte appearance), and the layers were arranged ±45° to the longitudinal direction of the laminates. The matrix material was bisphenol A-based epoxy resin. According to the ASTM D5868-01 [17] (American Standard Test Method for Lap Shear Adhesion for Fiber-Reinforced Plastic (FRP) Bonding), the laminates were cut into 101.6 mm × 25.4 mm × 2.5 mm pieces using a high-precision computer numerical control (CNC) machine.

The dimensions of Ni plates were 101.6 mm × 25.4 mm × l.5 mm. The sandblasting technology was used to prepare the surface. The abrasive material used in the process was 80# silicon carbide. The sandblasting angle was 90° (vertical injection), the pressure was 0.5–0.6 MPa, and the time was 10–20 s. The distance of the nozzle from the treated surface was 20 cm. The area of the treated surface was 25.4 mm × 25.4 mm, which corresponds to the bond line.

The preparation of surfaces of CFRP laminates was conducted according to ASTM D2093-03 [18]. In order to remove the glossy finish and all traces of dirt, grease, mold release or other contaminants from the bonding surfaces, CFRP laminates were first wiped with rayon balls dipped with acetone and sanded with 240# sandpaper in the longitudinal direction, then wiped by a clean dry cloth and finally wiped by rayon balls dipped with acetone again at room temperature. The sandblasted Ni plates were first cleaned by an ultrasonic cleaner, then wiped by rayon balls dipped with acetone and finally dried with a clean cloth.

### 2.2. Adhesive Bonding

**Figure 1:**
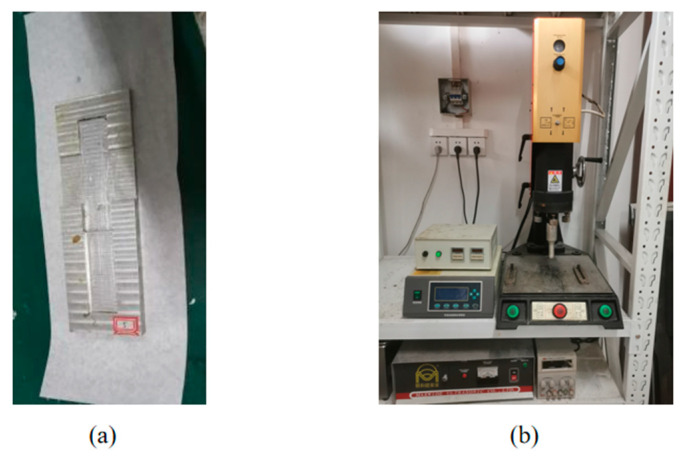
The fixture (**a**) and ultrasonic workbench (**b**).

The bonding operations were conducted according to ASTM D5868-01. The fixture, made of 7075 aluminum alloy, was used to prepare the joints, as shown in Figure 1a. In the bonding process, a CFRP laminate was first placed in the lower cavity of the fixture, then the epoxy adhesive was applied evenly on the CFRP laminate surface and finally a sandblasted Ni plate was correctly placed in the upper cavity. The upper cavity was 0.76 mm higher than the lower one and the Ni plate was tightly pressed by a heavy block; thus, the thickness of the bond line was ensured by the fixture to keep the consistency. A small amount of the adhesive was allowed to overflow from the bond line to ensure the sufficiency, and the additional adhesive was removed with a plastic spatula. After that, joints were randomly divided into experimental and reference groups. Seven samples of bonded joints were prepared in each group; two of them were prepared for cross-section characterization, and the others were prepared for the tensile tests. Ultrasonic vibration was applied in the experimental group but not in the reference group, and other operations remained the same for the two groups. During the ultrasonic process, the Ni plates were fixed, and the pressure was applied with the heavy block, weighing approximately 1 kg, to guarantee the adhesive layer thickness and restrict the relative displacement of the joints. After the ultrasonic treatment, all the joints were placed in an oven, where the adhesive was rapidly cured at 82 °C for 1 h. In order to allow the adhesive to fully cure and cool to room temperature, the samples were kept at room temperature for at least 12 hours. After curing, natural cooling and removal from the fixture, the samples were ready for testing. The actual lengths of the adhesive joints after the curing and demolding process were 177.8 mm, and their actual thicknesses were 4.76 mm. 

### 2.3. Ultrasonic Process

An ME-1800 vertical ultrasonic workbench, manufactured by MAXWIDE ULTRASONIC Co., Ltd. (No. 2, Minghe Road, Beiguan village, Malu Town, Jiading District, Shanghai, China), was used, as shown in Figure 1b. It consists of an ultrasonic generator, ultrasonic transducer, horn, sonotrode and pedestal, and all these components were manufactured by MAXWIDE. The ultrasonic generator converts household electricity into a high-frequency signal to drive the ultrasonic transducer, by which the signal is converted into mechanical vibration. The vibration is then amplified by the horn and transmitted to the sonotrode.

**Figure 2:**
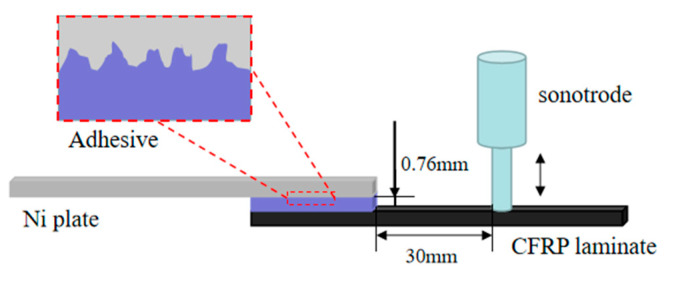
The schematic of the ultrasonic process.

In the ultrasonic process shown in Figure 2, a joint placed in the fixture was placed on the pedestal, and the sonotrode was pressed on the CFRP laminate to apply vibration. The distance between sonotrode and the leading edge of the Ni plate was 30 mm, and the vibration was transmitted to the adhesive layer through the CFRP laminate. In order to reduce excessive thermal effect, an intermittent mode of 2 s on and 1 s off was applied, and the total time of the ultrasonic process was 48 s. The parameters of the ultrasonic process are shown in Table 1.

### 2.4. Characterization

#### 2.4.1. Tensile Testing

The tensile test was performed to measure the shear strength of the bonded joints. According to ASTM D5868-01, the test was carried out using a universal tensile testing machine (SANS CMT5205 manufactured by MTS SYSTEMS (CHINA) Co., Ltd., Beijing, China); the parameters of the machine are listed in Table 2. The test was performed at room temperature and at a relative humidity of 50%. The beginning force of the test was 5 N. Sampling time interval was 0.01 s. The tensioning time was approximately 60 s. During the test, the speed was set as 2 mm/min. The peak loads were recorded, and the shear strength was calculated by the following equation:

(1) $$\tau =\frac{F}{B\times L}$$

where τ is the shear strength, F is the peak load, and B and L are the length and width of the bond line, respectively. Two aluminum alloy plates were placed on both ends of the sample to eliminate the influence of the bending moment. The thickness of the aluminum alloy plate on the CFRP laminate was 1.5 mm, and that of the other plate was 2.5 mm.

#### 2.4.2. Cross-Section Morphology

To study the mechanism of the strengthened bonding, SEM and EDS mapping were used to analyze the cross-section morphology of the joints. The bonded joints were cut into blocks of 5 mm × 5 mm × 4.76 mm, and then the cross-sections were polished. In order to improve the conductivity of the interface, it was coated with gold using an Oxford Quorum SC7620 sputter coater. The sputtering time was 90 s at a current intensity of 10 mA. Subsequently, the morphology and EDS mapping observations were performed using TESCAN MIRA LMS SEM. The acceleration voltage was 3 kV for the morphology photographing and 15 kV for the EDS mapping, and the detector was an SE2 secondary electron detector.

#### 2.4.3. Surface Morphology

Surface morphology was characterized using atomic force microscopy (AFM). Samples were taken from the pristine Ni plates, sandblasted Ni plates and failure joints of the experimental and reference groups after the tensile test. The samples were first cleaned using an ultrasonic cleaner for 5 min before the characterization. The morphology was scanned with a Bruker Dimension Icon AFM instrument with a scanning range of 5 μm × 5 μm.

## 3. Simulation

### 3.1. Modeling

The accuracy and rationality of the model have a great influence on the subsequent simulation process. The bonding model consisted of the CFRP matrix [19], Ni plate surface and uncured adhesive. Materials Studio 2019 was used to build the three parts, which were then combined to obtain the simulation model. After optimization and relaxation, the relaxed model was obtained.

#### 3.1.1. Adhesive Layer Modeling

**Figure 3:**
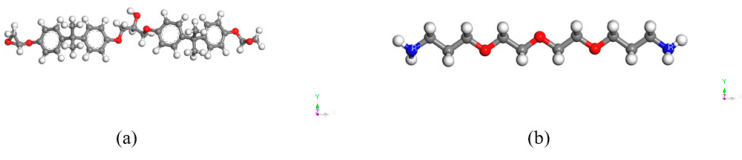
Molecular configurations of (**a**) bisphenol A diglycidyl ether-based epoxy resin and (**b**) a polyether diamine as curing agent.

The molecular configurations of the curing agent and epoxy resin shown in Figure 3 were built as primitive units for the adhesive layer. The main components of the adhesive were the epoxy and curing agent. The molecular configurations were constructed using Visualizer [20]. Hydrogenation and molecular arrangement were realized using the function of Adjust Hydrogen and Clean.

**Figure 4:**
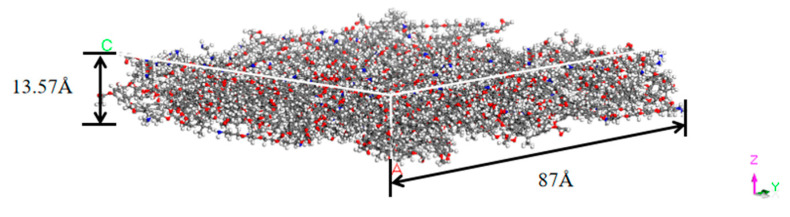
The model of the adhesive layer.

The adhesive layer model shown in Figure 4 was produced using the Calculation function in the Amorphous Cell module. The top and bottom surfaces were set as Confined Layer to limit the overflow of the adhesive molecules, and the other surfaces of the box were the periodic surfaces. The number of the epoxy molecules was set as 100, and the number of the curing agent molecules was 50. The density was set to 1.16 $g/{\mathrm{cm}}^{3}$ according to the adhesive data. To facilitate the calculation of the ultrasonic amplitude, the thickness of the model was set to 13.57 Å. To ensure the adhesive density, the side length (square) of the model was automatically calculated according to the density, and it was 87 Å.

#### 3.1.2. Matrix Modeling

**Figure 5:**
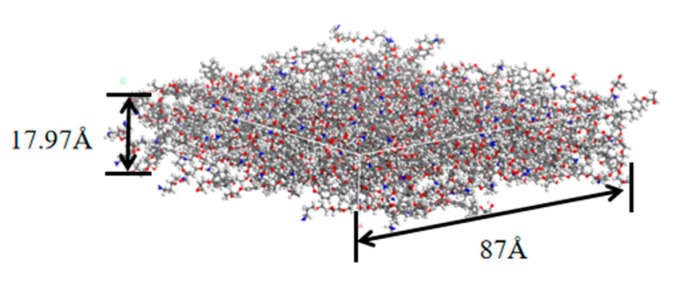
The model of the CFRP matrix.

The matrix of the CFRP is a resin cured by epoxy and curing agent in a number ratio of 2:1. The epoxy is bisphenol A diglycidyl ether type epoxy resin (DGEBA), and the curing agent is a polyether diamine (PEDA). The molecular configurations were constructed as described in the above section. The model before curing was built using the Calculation function, and the density was set to 1.10 $g/{\mathrm{cm}}^{3}$. Too few molecules might cause difficulty in achieving specified conversion, so the numbers of DGEBA and curing agent in the model were set to 200 and 100, respectively. After sufficient relaxation, the epoxy was crosslinked using the method of Wu et al. [21], which was implemented using a Perl script. In the crosslinking process, the reaction radius range was set as 3–11 Å, and the increase was 0.5 Å per step. The side length (square) of the model was set to 87 Å, which was the same as for the adhesive model. After the process, the CFRP matrix model of 87 Å × 87 Å × 17.97 Å was obtained with a crosslinking ratio of 88.25%, as shown in Figure 5.

#### 3.1.3. Ni Plate Modeling

**Figure 6:**
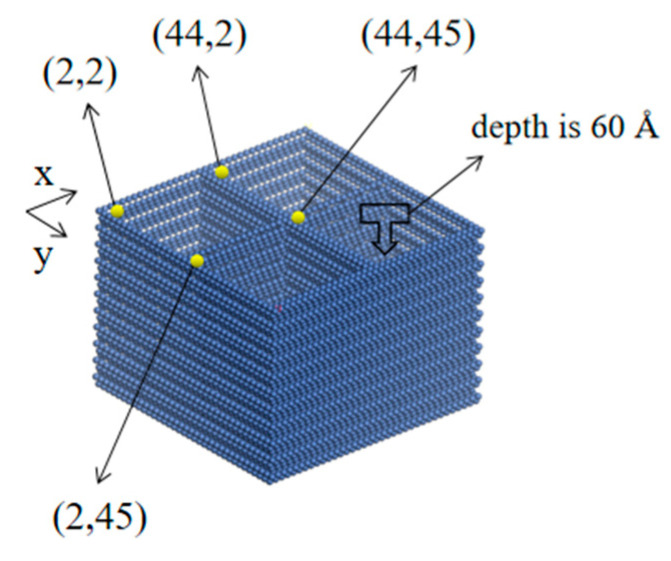
The model of the Ni plate surface.

The minimum energy section (1,1,1) of the nickel crystal cell was chosen as the surface of the Ni plate model. The minimum energy section was cleaved with a thickness of 63 Å, and then the cleaved plane was expanded to 87 Å × 87 Å using the Supercell function to obtain the initial Ni plate model of 87 Å × 87 Å × 63 Å. Afterward, four square pits were created on the surface of the model; the size of the pits was 4 nm × 4 nm. To observe the permeation of the adhesive during the simulation, the depth of the pits should be as large as possible. However, excessively large depth might cost much more simulation time. In this study, 60 Å was taken. The coordinates (unit: nm) of the four pits were (2,2), (44,2), (2,45) and (44,45), respectively. The established Ni plate model is shown in Figure 6.

#### 3.1.4. Bonding Interface Modeling

**Figure 7:**
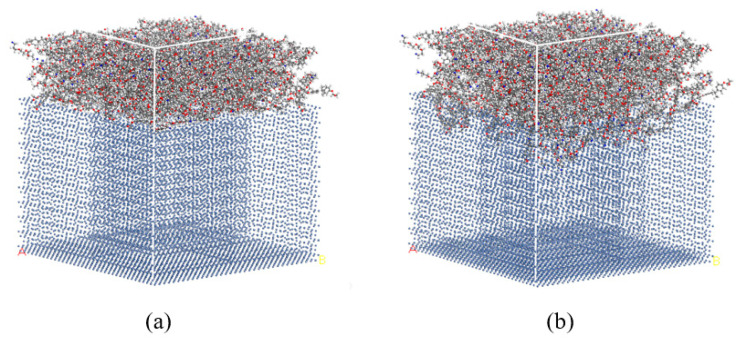
(**a**) The initial and (**b**) the relaxed bonding model.

The matrix, adhesive layer and Ni plate were combined to form a sandwich structure using Build Layers. In order to reduce the calculation time, the part of the matrix model was deleted if the coordinate Z > 90 Å, and that between 85 Å and 90 Å was set as the vibration layer. The Ni plate was fixed in the experiment, so the Ni model was constrained in the simulation. The bonding model is shown in Figure 7a.

Geometry optimization and relaxation were needed to stabilize the model; otherwise, large energy deviation or even dynamics error might occur in the simulation. The functions of Dynamics and Geometry Optimization in the Forcite module were used to relax and optimize the model, which could improve the accuracy and reduce deviation in subsequent calculations. The functions of Nose thermostat and NVT ensemble were used in the relaxation to keep the atom number, volume and temperature unchanged. COMPASS [22] was applied as the force field. Since the initial state of the model was not specified, the initial velocity was set as Random. Geometry optimization was carried out at both the beginning and the end of the relaxation process with the method of Smart, and the force field was consistent with that in the relaxation. The relaxed model is shown in Figure 7b.

### 3.2. Simulation Parameters

Equations (2)–(4) were prepared by us to calculate the parameters of the ultrasonic process simulation according to the size of the samples in the experiments and the bonding interface model established. The actual amplitude of the vibration ($A_{act}$) was 56 μm, and the actual thickness of the bond line ($T_{act}$) was 760 μm. The data are based on the ultrasonic process in Section 2.3. In order to satisfy the geometric similarity, the proportion of the amplitude ($A_{mod}$) to the adhesive thickness ($T_{mod}$) in the model should be the same as that of the experimental samples, as follows:

(2) $$\frac{A_{act}}{T_{act}}=\frac{A_{mod}}{n\times T_{mod}}$$

where *n* represents the multiple of the amplitude in the simulation. If *n* = 1 and 2.5, the amplitude is 1 and 2.5 times the experimental one, respectively.

Reynolds number is the ratio of inertia force to viscous force, which reflects the effect of fluid viscosity. Equal Reynolds numbers indicate the viscous similarity of two flow phenomena. The formula is expressed as follows:

(3) $$Re=\frac{v_{mod}d_{m}}{\mu }=\frac{v_{act}d_{p}}{\mu }$$

where $d_{p}$ represents the characteristic size of pits in the experiment. According to the SEM images, the size of the actual pits was 1 μm. $d_{m}$ represents the size of pits in the model, which is 4 nm. $v_{mod}$ and $v_{act}$ are ultrasonic velocities in the model and experiment, respectively. μ is the viscosity of the adhesive. The velocity ratio can be calculated by substituting the data into Equation (3), which turns out to be $v_{mod/v_{act}}=250$.

For two systems satisfying kinematic similarity, the velocity direction at a corresponding point is the same and the magnitude is proportional. The velocity is determined only by the vibration according to the following:

(4) $$C_{v}=\frac{v_{mod}}{v_{act}}=\frac{C_{T}}{C_{t}}=\frac{T_{mod}/T_{act}}{t_{mod}/t_{act}}$$

where $C_{T}$ and $C_{t}$ are the ratios of thicknesses and times, respectively. The calculated results of the parameters are shown in Section 3.3.

### 3.3. Ultrasonic Process Simulation

**Figure 8:**
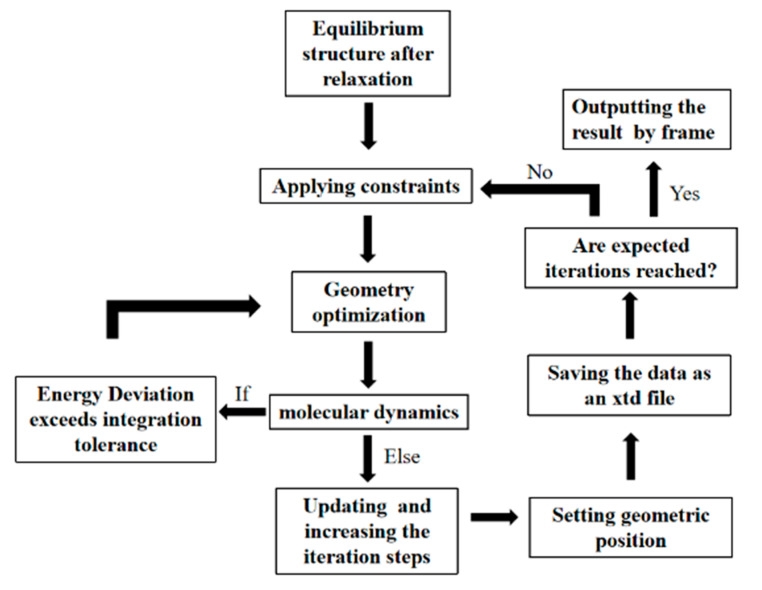
The flow chart of ultrasonic script.

A Perl script was used to apply ultrasonic vibration with different amplitudes and frequencies. The flow chart is shown in Figure 8. The parameters of the molecular dynamics simulation are given in Table 3.

The simulation was divided into two parts. In Part 1, the Perl script was set to simulate the ultrasonic process with the same amplitude (*n* = 1) but different frequencies (20 kHz, 25 kHz and 30 kHz). The simulation parameters shown in Table 4 were calculated by Equations (2)–(4) in Section 3.2 according to the dimensions of the samples in the experiments and the bonding interface model established. In Part 2, the Perl script was set to simulate the ultrasonic process with the same frequency (20 kHz) but different amplitudes (1 Å and 2.5 Å). Boundaries and constraints are consistent within the two parts.

## 4. Results and Discussion

### 4.1. Tensile Test Results

**Figure 9:**
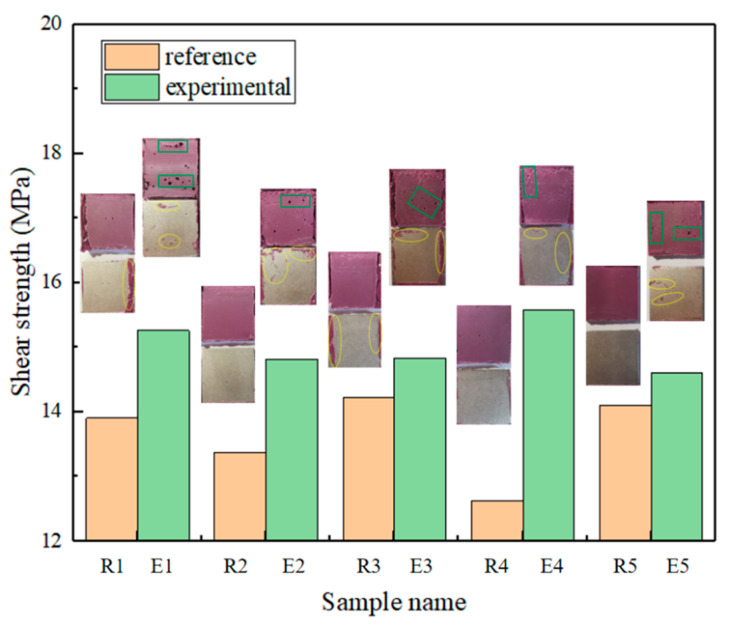
Shear strength and failure of adhesive joints in the experimental and reference groups.

The shear strength of experimental and reference groups is shown in Figure 9 and Table 5. Both the experimental and reference groups consisted of five repetitive samples, and the samples were named E1, E2, E3, E4 and E5 and R1, R2, R3, R4 and R5. The average bonding strength of the experimental group and the reference group was 15.02 MPa and 13.64 MPa, respectively. The standard deviation from the mean value was 0.35 and 0.58, respectively. The bonding strength of the experimental group was increased by 10% compared to that of the reference group.

The failure adhesive joints after the tensile test are shown in Figure 9. The failure modes of the joints were classified according to ASTM D5573-99 [23]. In the experimental group (E1, E2, E3, E4 and E5), all the five joints showed mixed failure mode, including adhesive failure and thin-layer cohesive failure. The percentage of the adhesive failure was 64%, 56%, 44%, 60% and 76%, respectively. Residual adhesive could be observed on the sandblasted surfaces (marked by ellipses). The surfaces of the failed adhesive layer were relatively rough, and many holes could be observed, as marked by rectangles. The reason for the appearance of the holes was that the joints with the ultrasonic treatment were more tightly bonded, and thus the adhesive layers were torn during the tensile tests. In the reference group (R1, R2, R3, R4 and R5), mixed failure mode was shown in two samples (R1 and R3), and the percentage of the adhesive failure was 76% and 60%. Adhesive failure occurred at the adhesive/Ni interface of the remaining three samples. Residual adhesive could hardly be observed on the sandblasted surfaces. In comparison with the failure of the experimental group, the surface of the failed adhesive layer was much smoother. The results indicated that the ultrasonic treatment promoted the adhesion of the interface.

### 4.2. Cross-Section Morphology Analysis

**Figure 10:**
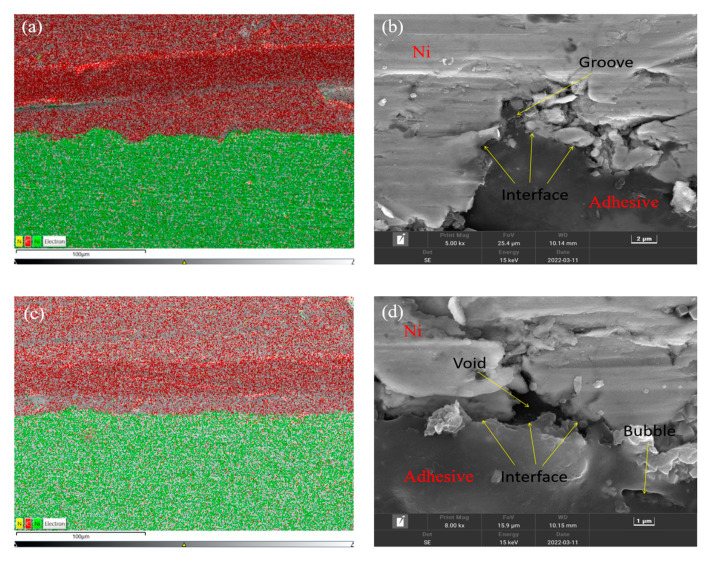
EDS mapping images of the interface (**a**) with and (**c**) without the ultrasonic treatment, and SEM images of the interface (**b**) with and (**d**) without the ultrasonic treatment.

The EDS and SEM images of the interface with the ultrasonic treatment are shown in Figure 10a,b, and those without the treatment are shown in Figure 10c,d. In the EDS images, the distributions of the elements C and Ni are indicated by red and green dots, respectively. The interface could be identified by the distributions of C and Ni. The adhesive was determined by the distribution of C, for it is the main element of the adhesive chemical composition. In the SEM images, it can be seen that the Ni plate surface was bumpy after the sandblasting treatment. Voids were produced owing to the poor permeation of the adhesive into the pits as shown in Figure 10d. As shown in Figure 10b, after the ultrasonic treatment, the pits at the interface were significantly filled with the adhesive, and the defects were fewer. The results indicated that the permeation of the adhesive was much better when using the ultrasonic application and more adhesive penetrated into the pits on the sandblasted surface. The penetrated adhesive formed anchors at the interface, and the actual bonding area was also increased.

### 4.3. Surface Morphology Analysis

**Figure 11:**
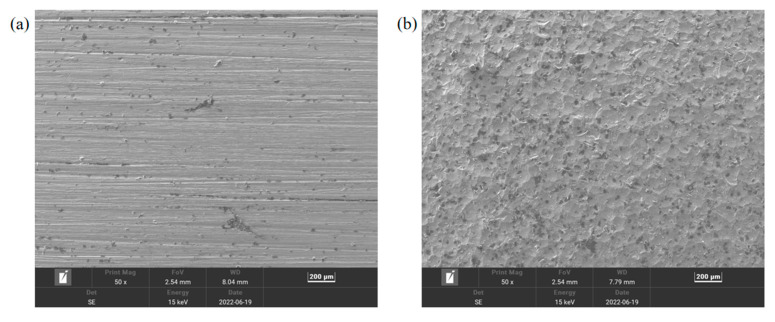
SEM images of the surfaces of (**a**) pristine Ni plate and (**b**) sandblasted Ni plate.

The SEM images of the surfaces of the pristine Ni plate and the sandblasted Ni plate are shown in Figure 11. Because hard particles were blasted on the surface, the sandblasted surface exhibited deep cavities and cracks spreading over the whole surface. Compared to the surface of pristine Ni plate, sandblasting increases the surface roughness of Ni plate, which is conducive to the formation of interface interlocking.

**Figure 12:**
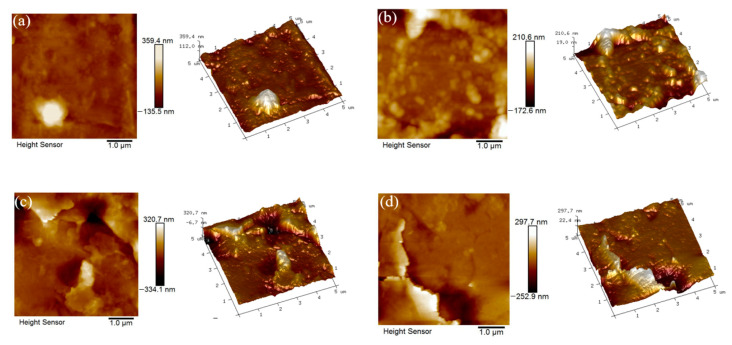
AFM images of the Ni plate (**a**) without and (**b**) with sandblasting and the failure surface of the joints from (**c**) experimental and (**d**) reference groups after the tensile test.

The AFM images of the Ni plates are shown in Figure 12, and the measured data are listed in Table 6. The roughness parameters *Rq* and *Ra* represent the root mean square deviation and arithmetic mean deviation of the data according to ASME B46.1 [24]. *Ra* is used as the parameter for evaluating the surface roughness of porous materials. It is defined as the average absolute deviation from the mean line over one sampling length of the surface roughness, and it is calculated as follows [25]:

(5) $$Ra=\frac{1}{N_{x}N_{y}}\sum_{i=1}^{N_{x}}\sum_{j=1}^{N_{y}}\left|z\left(i,j\right)-z_{mean}\right|$$

where

(6) $$z_{mean}=\frac{1}{N_{x}N_{y}}\sum_{i=1}^{N_{x}}\sum_{j=1}^{N_{y}}z_{ij}$$

$N_{x}$ and $N_{y}$ represent the number of points on the *X* and *Y* axes, respectively.

*Rq* is the standard deviation of the surface height distribution, and it is defined as follows:

(7) $$Rq=\sqrt{\frac{1}{N_{x}N_{y}}\sum_{i=1}^{N_{x}}\sum_{j=1}^{N_{y}}{\left(z\left(i,j\right)-z_{mean}\right)}^{2}}$$

Figure 12a shows the surface of the pristine Ni plate, which is a flat surface with small *Rq* and *Ra* of 49.0 nm and 29.3 nm. Figure 12b shows the surface of the sandblasted Ni plate. A number of bumps appeared and the surface was no longer flat. Compared to the 3D image of the pristine Ni plate, many micro pits and bulges were formed on the surface by the sandblasting. *Rq* and *Ra* were larger at 79.0 nm and 54.3 nm. Figure 12c shows the failure surface morphology of the reference group. The roughness parameters *Rq* and *Ra* were 76.9 nm and 52.6 nm, respectively, which were slightly smaller than those of Figure 12b. Some pits were filled with the adhesive. Figure 12d shows the surface morphology of the experimental group. It can be seen that more pits were filled with the adhesive, which made the surface smoother than that in Figure 12c. The results showed that the surface with the sandblasting treatment was rougher than that of the pristine material. Furthermore, the permeation of the adhesive in the pits was improved owing to the ultrasonic action.

### 4.4. Analysis and Discussion of the Simulation

#### 4.4.1. Different Frequencies

**Figure 13:**
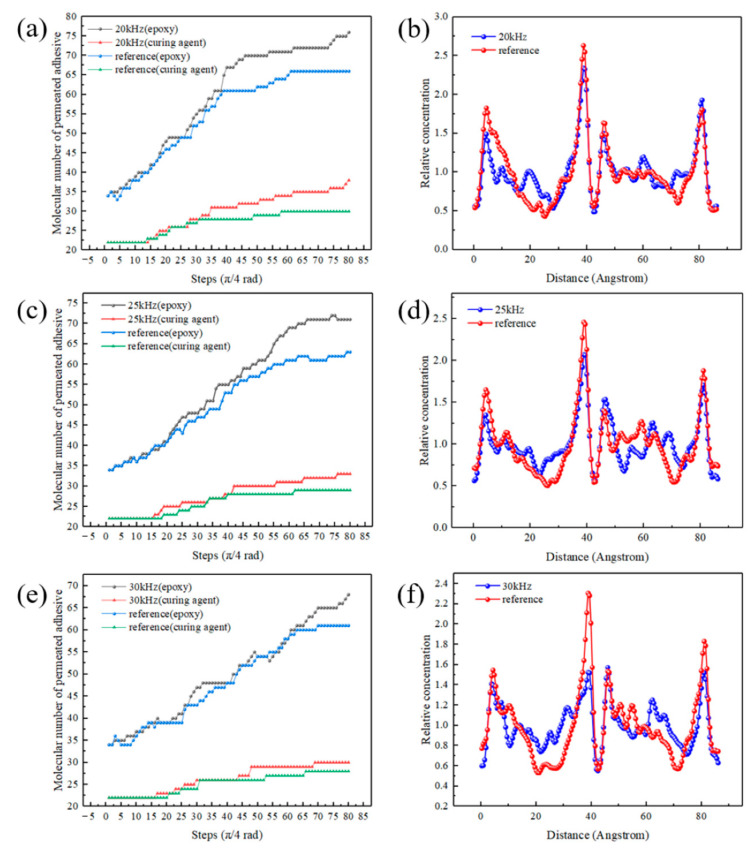
Molecular numbers of permeated adhesive under the ultrasonic action of (**a**) 20 kHz, (**c**) 25 kHz and (**e**) 30 kHz and concentration profiles of the final states of (**b**) 20 kHz, (**d**) 25 kHz and (**f**) 30 kHz.

Figure 13a,c,e show the number of adhesive molecules (molecular number) that penetrated into the micro pits in the simulation. The Y axes of the Figure 13a,c,e represent the molecular numbers of permeated adhesive. The criterion was that the Z coordinate of the molecular centroid was smaller than that of the Ni surface (Z ≤ 63 Å). Figure 13a,c,e show permeation speed was rapid at the initial stage. After several cycles, the permeation gradually weakened with the tendency of reaching a plateau. The molecular number of the permeated adhesive in the experimental group was higher than that in the reference group, which directly proved that the ultrasonic vibration could promote the adhesive permeation. With the increase in frequency, the permeation became slower. The molecular numbers of the permeated adhesive increased by 10%, 8% and 7%, respectively, with the frequency of 20 kHz, 25 kHz and 30 kHz after 10 cycles.

The function of Concentration Profile was used to evaluate the concentration distribution of the adhesive. Figure 13b,d,f show the concentration profiles. The *Y* axes of Figure 13b,d,f represent the relative concentration of permeated adhesive, and the *X* axes represent the distance along the chosen direction (1,0,0). The relative concentration is a dimensionless quantity; a value of 2 means that there is twice the number of atoms in the layer than if all atoms were distributed homogeneously across the system. The total number of atoms across all layers is equal to the number of atoms in the entire system. So, the sum of the relative concentrations of all layers is equal to the number of layers. Relative concentration could be explained by Equation (8). During the calculation, the model was first divided into 170 layers along the (1,0,0) (*X*-axis) direction, and then the relative concentration was calculated. The relative concentration of a layer n ($C_{n,relative}$) in the *X*-axis direction was calculated by the following equation:

(8) $$C_{n,relative}=\frac{N_{\left[\left(n-1\right)\times width\leq x\leq n\times width\right]}}{N}\times Z$$

where $N_{\left[\left(n-1\right)\times width\leq x\leq n\times width\right]}$ is the number of atoms whose coordinates are within the range, N is the total number of atoms in the adhesive and width is the thickness of one layer. Z is the total number of layers, which is 170, so that the sum of all the relative concentrations satisfies

(9) $$\sum_{n=1}^{Z}C_{n,relative}=Z$$

The peak value near the coordinate *X* = 42 Å was due to the aggregation of the adhesive molecules, because the molecules were adsorbed by the sidewalls of the pits there. Variances of the concentration profiles were calculated, and the results are listed in Table 7. The variances of 20 kHz, 25 kHz and 30 kHz for the experimental group were 0.0549, 0.0411 and 0.0349, respectively, and those of the reference group were 0.0844, 0.0560 and 0.0514, respectively. Through the comparison of the profiles and variances, it can be seen the concentration difference of the adhesive molecules was reduced at the bonding interface, and the adhesive was better dispersed for the experimental group. The ultrasonic application of different frequencies promoted the permeation of the adhesive into pits.

#### 4.4.2. Different Amplitudes

**Figure 14:**
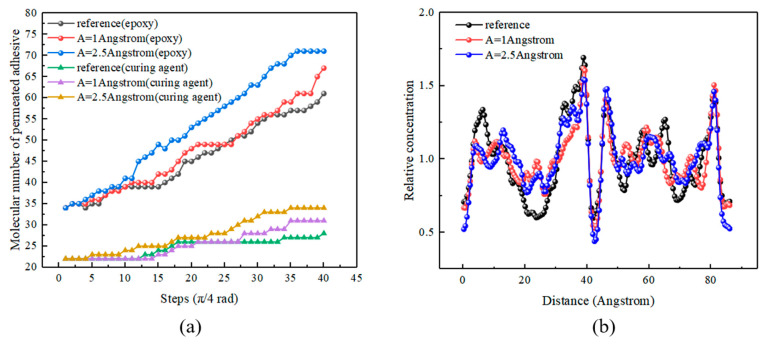
Molecular numbers of permeated adhesive (**a**) and concentration profiles (**b**) of the final states of the simulations with different amplitudes.

Figure 14a shows molecular numbers of the permeated adhesive with different amplitudes. With the increase in amplitude, the numbers rose. After five cycles, the numbers of the permeated adhesive of A = 1 Å and 2.5 Å increased by 6% and 10%, respectively, compared to that of the reference group. In addition, Figure 14a shows that an appropriate increase in ultrasonic amplitude played a positive role in strengthening the interlocking. Similarly, the (1,0,0) (*X*-axis) direction was selected to calculate the concentration profile, and the results are shown in Figure 14b. The variances of concentration profiles were calculated and are listed in Table 8. The results showed that the increase in amplitude improved the uniformity and permeation of the adhesive layer.

#### 4.4.3. Average Value of Kinetic Energy

**Figure 15:**
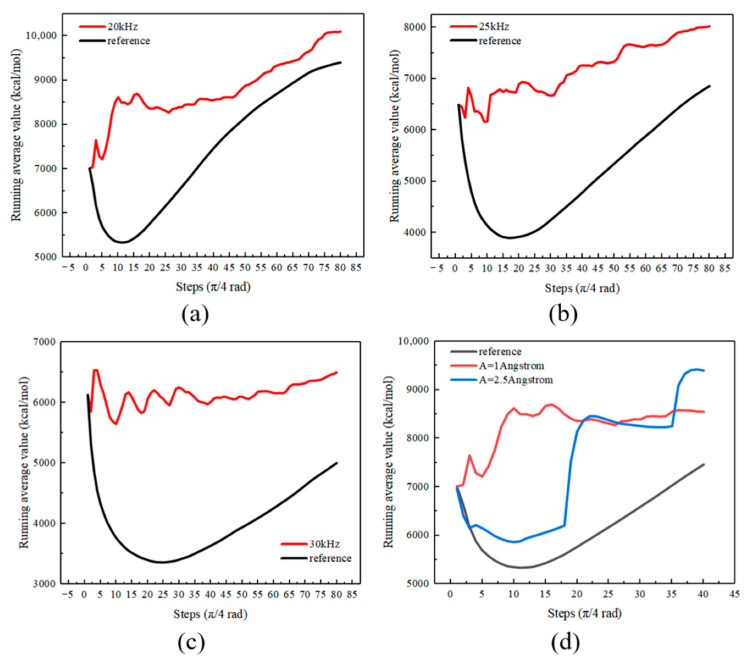
Average kinetic energy of adhesive molecules in the simulation of (**a**) 20 kHz, (**b**) 25 kHz, (**c**) 30 kHz and (**d**) different amplitudes.

The average value of kinetic energy was calculated using the Total Kinetic Energy function. The average value of kinetic energy was useful in evaluating the ultrasonic effect on the movement of adhesive molecules within a specific time period. Each calculated data point of the average kinetic energy is the cumulative average of the selected property for all selected steps between the first and the current step. Figure 15 shows the average kinetic energy profiles. The duration of one step is one-eighth of an entire cycle. The formula of the average value of kinetic energy for a step n (i.e., at the time of *n*/(8 × frequency)) is

(10) $$E_{k,n,average}=\frac{\sum_{i=1}^{n}E_{k,i}}{n}$$

where $E_{k,i}$ is the total kinetic energy of the model in a step i.

It can be seen that the ultrasonic vibration with different frequencies and amplitudes increased the total kinetic energy of the model because of enhanced molecular friction, and the movement of adhesive molecules was promoted. Larger molecular kinetic energy indicates higher adhesive temperature, with a beneficial improvement of adhesive fluidity for the experimental group when compared to the reference one. Therefore, the permeation of the adhesive into the pits was facilitated.

## 5. Conclusions

In this study, ultrasonic vibration was employed during the bonding process of obtaining CFRP/Ni joints. The cross-section morphology of the joints was investigated using EDS mapping and SEM. The surface morphology with different treatments was scanned by AFM, and the bonding strength was determined by tensile tests. Then, the microscopic model of the ultrasonic process was designed using the software package Materials Studio 2019 to study the strengthening mechanism. The conclusions are as follows:

(1) Sandblasting treatment could be used to adjust the surface roughness, and ultrasonic vibration-assisted adhesive bonding could not only increase the permeation of the adhesive, but also effectively reduce the defects such as bubbles and voids to form a compact interface.
(2) More anchors between the surface of the adherend and adhesive layer were formed as a result of the ultrasonic action. The shear strength of the bonded joints with the ultrasonic treatment increased by 10% compared to that without the treatment.
(3) According to the molecular simulation, the ultrasonic action could improve the permeation and uniformity of adhesive at the interface. The molecular numbers of permeated adhesive increased by 10%, 8% and 7% with the frequency of 20 kHz, 25 kHz and 30 kHz, respectively, after 10 cycles, and those increased by 6% and 10% with the amplitude of 1 Å and 2.5 Å, respectively, after 5 cycles.
(4) The molecular kinetic energy of the model rose under the ultrasonic action because of enhanced molecular friction, so the adhesive fluidity was improved, facilitating the permeation.

## Figures and Tables

**Table 1:** Specifications of the ultrasonic process.

Parameter	Specification
Equipment model	ME-1800
Operating mode	Intermittent
Input voltage	220 V/AC
Frequency	20 kHz
Amplitude	56 μm
Method	2 s on and 1 s off
Total time	48 s
Cooling mode	Air cooling
Size of workbench	540 × 400 × 1050 mm

**Table 2:** Specifications of the universal tensile testing machine.

Parameter	Specification
Model	CMT5205
Maximum force	200 kN
Voltage	380 V
Power	2.9 kW
Level of accuracy	0.5%
Serial number	11511029

**Table 3:** Specifications of molecular dynamics simulation.

Parameter	Specification
Forcefield	COMPASS
Thermostat	Nose
Ensemble	NVT
Initial velocities	Use current
Temperature	298 K

**Table 4:** Specifications of ultrasonic vibration simulation.

Experiment	Simulation					
Frequency	Frequency	Amplitude	Period	Duration/Step	Radians/Step	Cycle
20 kHz	2800 GHz	1 Å	358 fs	44.8 fs	π/4	10
25 kHz	3500 GHz	1 Å	286 fs	35.7 fs	π/4	10
30 kHz	4200 GHz	1 Å	238 fs	29.7 fs	π/4	10

**Table 5:** Shear strength of the two groups.

Group	Sample Name	Failure Load (N)	Shear Strength (MPa)	Average Strength (MPa)	Standard Deviation
Experimental	E1	9847.57	15.26	15.02	0.35
	E2	9563.31	14.82		
	E3	9568.42	14.83		
	E4	10,046.00	15.57		
	E5	9425.99	14.61		
Reference	R1	8968.08	13.90	13.64	0.58
	R2	8626.55	13.37		
	R3	9176.01	14.22		
	R4	8149.63	12.63		
	R5	9099.74	14.10		

**Table 6:** Measured data of the surface roughness explored via AFM.

	*Rq*	*Ra*
Pristine Ni plate	49.0 nm	29.3 nm
Sandblasted Ni plate	79.0 nm	54.3 nm
Failure surface from reference group	76.9 nm	52.6 nm
Failure surface from experimental group	60.4 nm	37.8 nm

**Table 7:** Variances of concentration with different frequencies.

Frequency	Experimental Group	Reference Group
20 kHz	0.0549	0.0844
25 kHz	0.0411	0.0560
30 kHz	0.0349	0.0514

**Table 8:** Variances of concentration with different amplitudes.

Amplitude	Variance
0 Å	0.0918
1 Å	0.0642
2.5 Å	0.0370

## Data Availability

Data sharing not applicable.
